# Ventanillas de Salud: A Collaborative and Binational Health Access and Preventive Care Program

**DOI:** 10.3389/fpubh.2017.00151

**Published:** 2017-06-30

**Authors:** Maria Gudelia Rangel Gomez, Josana Tonda, G. Rogelio Zapata, Michael Flynn, Francesca Gany, Juanita Lara, Ilan Shapiro, Cecilia Ballesteros Rosales

**Affiliations:** ^1^Secretaría de Salud de México, Mexico City, Mexico; ^2^Instituto de Mexicanos en el Exterior, Mexico City, Mexico; ^3^Mexico Section, US Mexico Border Health Commission, Tijuana, Mexico; ^4^National Institute for Occupational Safety and Health, Washington, DC, United States; ^5^Memorial Sloan Kettering Cancer Center, New York, NY, United States; ^6^VDS Program, Mexico City, Mexico; ^7^Ventanillas de Salud, Mexico City, Mexico; ^8^Mel and Enid Zuckerman College of Public Health, University of Arizona, Phoenix, AZ, United States

**Keywords:** prevention programs, binational programs, border health, health-care access, collaborative programs

## Abstract

While individuals of Mexican origin are the largest immigrant group living in the U.S., this population is also the highest uninsured. Health disparities related to access to health care, among other social determinants, continue to be a challenge for this population. The government of Mexico, in an effort to address these disparities and improve the quality of life of citizens living abroad, has partnered with governmental and non-governmental health-care organizations in the U.S. by developing and implementing an initiative known as *Ventanillas de Salud*—Health Windows—(VDS). The VDS is located throughout the Mexican Consular network and aim to increase access to health care and health literacy, provide health screenings, and promote healthy lifestyle choices among low-income and immigrant Mexican populations in the U.S.

## Introduction and Background

The social determinants of health are the conditions in which people are born into, grow from, live in, and work including a wide range of other forces and governmental and social systems that shape daily life (1). In immigrant groups, the social determinants of health are a combination of these conditions between the country of origin and the destination where migrant populations will form new lives subjected to new governmental, social, and culturally established infrastructures.

The U.S. census estimated 33 million individuals of Mexican origin resided in the U.S. in 2010. Of the 11.2 million undocumented immigrants in U.S. (2), an estimated 5.9 million were from Mexico (3). Health disparities and limited access to care continue in this population (4–6). By 2012, 53% of immigrants from Mexico lacked health insurance (7). By contrast, DeNavas-Walt et al. (8) found that only 13% of U.S. born individuals were uninsured in 2012. Furthermore, 61% of those born in Mexico, who work and contribute to the U.S. economy (18–64 years of age) reported lacking health insurance. This situation is not limited to this age group, according to the Health Needs Survey conducted by the Mexican Consulate General in Los Angeles in 2004. This survey reported that 49% of children born to Mexican parents in the U.S. lacked health insurance, notwithstanding, the state of California, which offered free insurance for low-income children of age 18 and under (9).

In the U.S., many factors impede Mexican immigrants accessing health services. These barriers include the following: low socioeconomic status (4, 10), which is associated with educational attainment, occupation, and income (11); limited English speaking skills (3, 12–16); and immigration status (17–19). Moreover, place of residence and structural factors such as stigma, racism, and anti-immigrant policies further influence individual behaviors in accessing health services (20–22). Services offered in their preferred language are yet another factor (23), as well as family composition, such as mixed immigration status (22).

Given this background, in 2003, Mexico’s Ministry of Health created the Ventanillas de Salud (VDS) program as part of an unprecedented effort to use its Consular network to promote well-being in efforts to reduce health disparities among the Mexican diaspora in the U.S. (24–26). The VDS program aims to promote and enhance the health of Mexicans living abroad, particularly in the U.S. and offers an exceptional opportunity for partnerships with U.S. public health agencies (9). This article describes how the VDS program was created to address the most pressing health disparities within the immigrant Mexican population, the strategic alliances forged and the impact of this program. It further provides a description of the size and scope of the services offered through the VDS in 2013.

### VDS Program (27)

In 2003, a pilot project providing information on basic health issues and access to preventive care was introduced in San Diego, CA, USA. The purpose of this pilot was to gather information concerning the main health problems and access to health services for immigrants of Mexican origin. This pilot program continued in 2004 with the aim of establishing the groundwork for replicating the model within the Mexican Consular Network in the U.S. Following the pilot and two evaluation studies conducted in 2004, the VDS program established 11 additional VDS between 2004 and 2007 within the Mexican Consular Network. Between 2008 and 2011, an additional 38 VDS opened for a total of 50 affiliated with the Mexican consulates that work closely and in partnership with local health-care organizations. In 2012, Kansas City initiated a mobile unit and in 2013 New Jersey followed suit with a second mobile unit serving as the VDS outreach arm of their respective programs.

The VDS is the result of collaborative efforts between two federal agencies within the Mexican Government—Ministry of Health and Ministry of Foreign Affairs—working in partnership and cooperatively with U.S. governmental and non-governmental health-care organizations that provide the available and needed services. Currently, the government of Mexico provides monetary and in-kind resources to support the program in its entirety. These resources are in turn leveraged by the organizations, foundations, and local sponsors of each VDS. For example, federally qualified community health centers located throughout the U.S. provide primary and secondary prevention services on a sliding fee scale.

This network provides reliable information on health promotion and disease prevention, counseling, and referrals to available, affordable, and accessible services to the immigrant Mexican population in the U.S. The program is designed to increase access to health-care services and health insurance enrollment, for those qualified, and decrease use of emergency medical services, thereby improving the physical and mental health of immigrant Latinos (9).

The VDS program offers a standard package of services across the 50 consulates (defined according to the main health issues identified for the Latino population, adjusted in some particular situations), and guarantee a culturally and linguistically sensitive services allowing clients to fully comprehend the nature and importance of health care and utilization of the existing health-care infrastructure. The potential scope of the VDS is significant, since the Consular Network serves on average 1.5 million individuals annually through its 50 VDS.

The VDS is a unique model of binational collaboration linking Mexican Consulates, local health-care providers, and organizations dedicated to improving access to health-care services, providing a safe and secure environment for Mexican immigrants and their families living in the U.S. through information, health assessment, and health education.

The mission of the VDS is to provide disease prevention and health promotion services, which includes information about the medical and health insurance marketplace in Mexico and in the U.S., and referral to health-care services. It is up to the individual client to follow through in receiving needed services; although, a few of the VDS do provide navigators or health promotores (community health workers) that assist clientele in securing medical insurance from the marketplace, as well as tracking clients referred for specialized services to ensure they receive follow-up care. Upon written consent of the client, tracking is accomplished through direct phone calls or through emails by the health promotores to determine the status of the referral.

## Methods and Data

The data presented correspond to clients accessing services at the 50 VDS during 2013. Each client completed a standardized form upon accessing services at one of the 50 VDS. The standardized form included a series of questions on demographics, history of access to medical services, personal medical history, anthropometric measurements, services rendered, referrals made, screening tests, and results. This information was collected by health promotores at each of the 50 VDS. Health promotores receive training on data collection. The database was maintained in a secure file and was subsequently reported on a quarterly basis to the Ministry of Health of Mexico. Data obtained by the coauthors were de-identified. The aggregate data were analyzed using SPSS version 19 software to provide the descriptive analysis for this article. The project was submitted for approval by the ethics committee established by of the Mexico Section of the US-Mexico Border Health Commission.

## Results

The number of clients accessing services from the 50 VDS between January and December 2013 totaled 1,182,760, of which 225,058 (19%) were provided by the mobile VDS. A snapshot of VDS participants shows the greatest number of users is within the reproductive age, with 34% between ages of 36 and 45 followed by 26- and 35-year olds with 29%. In addition, 63% of VDS users are women while 37% are men. While the VDS program does not inquire about immigration status, demographics on country of origin show users to be predominantly from Mexico and Latin America followed by Caribbean countries and territories. Languages reported include Spanish, English, and indigenous languages to Mexico and Latin America.

**Figure 1:**
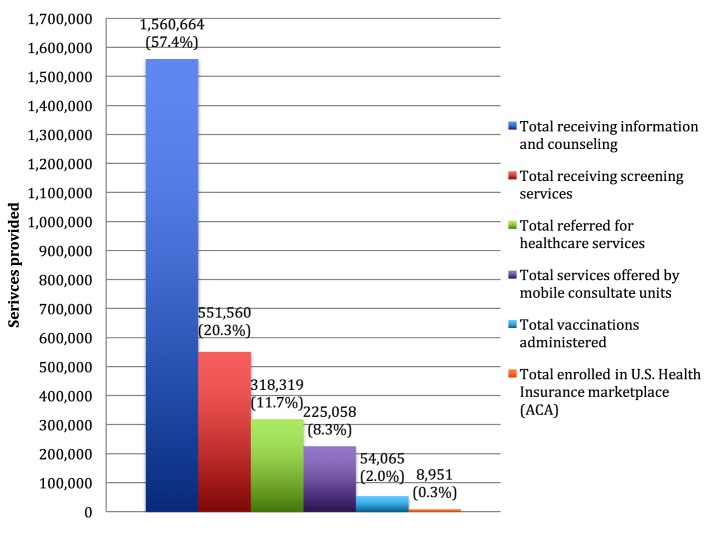
Number of services offered by the 50 Ventanillas de Salud (VDS) by type of service, 2013. Source: Indicators report of the VDS program (27). Period from January to December 2013. Number of Ventanillas per period: 50 January–March 2013, 52 April–June 2013, 52 July–September 2013, and 52 October–December 2013.

These same clients received 2,718,617 services; an average of 2.3 services per client. Services included educational information, service referrals to community health-care centers, screenings, and immunizations. In each of the VDS, on average 23.7 clients were serviced, ranging from 1,453 client visits made to the VDS in Anchorage, AK, USA compared to 13,423 visits made in Los Angeles, CA, USA. Of the total services offered, 57.4% involved information and referral. Educational health topic orientations by trained CHWs included diabetes, obesity, women’s health, hypertension, mental health, substance abuse, family violence, birth control, and health insurance (both the Affordable Care Act marketplace for eligible individuals and Mexico’s *Seguro Popular* program). Another 20.3% received screening for diabetes, hypertension, cholesterol, and HIV/AIDS (Figure 1).

A total of 116,468 blood glucose screenings were performed. Of these, 32% (31.9%) had high glucose levels. Of the 50,272 consumers measured for body mass index, half were found to be overweight or obese. Blood pressure screens totaled 14,997, of which 32.6% had a systolic blood pressure greater than 130 mmHg. Approximately 16,369 HIV blood screens were performed, and 1.6% found to be positive. The prevalence of sexually transmitted infections of those receiving testing equaled 3.7% (Table 1).

**Table 1:** Prevalence of the leading causes of morbidity detected in the migrant Mexican population at the *Ventanillas de Salud (VDS)*, January–December 2013.

Measurements	Orientation sessions	Assessments performed	Positive tests/high levels	Prevalence (%)
Blood glucose	195,162	116,468	37,176	31.9
Obesity and Overweight	126,185^a^	50,272	25,115	49.9
Blood pressure	124,255	124,997	40,829	32.6
Cholesterol	126,185^a^	17,496	3,998	22.9
HIV/AIDS	71,801^b^	16,369	266	1.6
STI	71,801^b^	4,619	169	3.7
Tuberculosis	1,989	399	21	5.3

*Source: VDS program-Indicator Report (27)*.

*Number of Ventanillas per period: 50 January–March 2013; 52 April–June 2013*.

*Blood pressure in accordance with the British Hypertension Society (BHS-IV), http://www.bhsoc.org/latest-guidelines/a/*.

*BMI definition (obesity and overweight): WHO, http://www.who.int/mediacentre/factsheets/fs311/es*.

*Blood glucose definition: WHO-99, http://www.who.int/diabetes/action_online/basics/es/index2.html*.

*52 July–September 2013; 52 October–December 2013*.

*^a^Includes counseling sessions on obesity prevention/metabolic syndrome/cholesterol*.

*^b^Includes counseling sessions on prevention of STI and HIV/AIDS*.

In addition, 63,755 clients of the VDS received orientations; 39.4% were referred for secondary and tertiary care for substance use. Of those receiving mental health counseling, 2.6% (56,368) were referred for additional services. A total of 318,319 clients were referred to community clinics for health-care services, and another 86,273 received information about available programs.

The VDS clients received health insurance program information. A total of 8,951 clients were enrolled in the U.S. health insurance marketplace. Health insurance enrollment is a service provided by some of the VDS; especially given the challenges of enrolling the Mexican population in the U.S. (Figure 1).

## Discussion

The *VDS* program (27) serves as a resource providing Mexican immigrants in the U.S. information, referral, and health education on promoting healthy lifestyles while living abroad. The mission of the VDS is reassuring the safety and health of immigrants and families where they work and live across the U.S.; thereby, realizing their full potential physically, mentally, and socially. The VDS program is an effort by the Mexican Government in collaboration with local U.S. Health Institutions, agencies, and non-governmental health organizations to address barriers and reduce disparities with the Mexican immigrants in accessing health care and meeting their medical needs.

Through this program, a significant number of services have been granted, which have enabled not only to guide and inform the community about the different conditions that affect the Latino population living in the U.S. but also to detect the presence of high levels of blood glucose, overweight, and obesity, HIV/AIDS, among other conditions. Referrals for follow-up and specialized care to community clinics is another important objective of this program as, through timely detection and referral to health services, the VDS program helps reduce emergency room visits and establish a medical home.

The impact of the VDS program is largely due to the support of partnerships with more than 500 agencies, both public and private, that impact and help improve the health of Mexicans who reside in the U.S. These partnerships have become a comprehensive preventive health-care safety-net model for Mexican communities abroad, making the *VDS* the main disseminator of programs for prevention and health promotion among Mexicans in the U.S., with the purpose of guaranteeing their constitutional right to health, regardless of the territory where they live. The VDS model has also been adopted by other Latin and Central American countries exploring ways to support the needs of their population living and working abroad.

Currently, the VDS program faces an important challenge to achieve long-term sustainability for its expanding programs, and this will depend, to a large extent, on the support and continued partnerships of health institutions in the U.S. To achieve this, it is essential to establish and strengthen alliances with key institutions, such as national institutes of health, Mexican migrant associations, local city and state governments, academic institutions and civil society organizations, among many others.

The VDS program continues to grow and expand its reach by creating new venues to resolve current health problems and maintain flexibility and adapt to local trends and health-care needs.

## Author Contributions

MG contributed to the initial drafting and analysis of data; JT, GZ, JL, MF, FG, IS, and CR contributed to review and revision of data analysis, interpretation, and discussion.

## Conflict of Interest Statement

The authors declare that the research was conducted in the absence of any commercial or financial relationships that could be construed as a potential conflict of interest.
